# Pseudo-Rosette-Forming Blastic Plasmacytoid Dendritic Cell Neoplasm

**DOI:** 10.4274/tjh.galenos.2020.2020.0141

**Published:** 2020-08-28

**Authors:** Praveen Sharma, Shano Naseem

**Affiliations:** 1 Department of Hematology, Post Graduate Institute of Medical Education and Research, Chandigarh, India

**Keywords:** Blastic plasmacytoid dendritic cell neoplasm, Pseudorosette, Bone marrow

## To the Editor,

**Figure 1:**
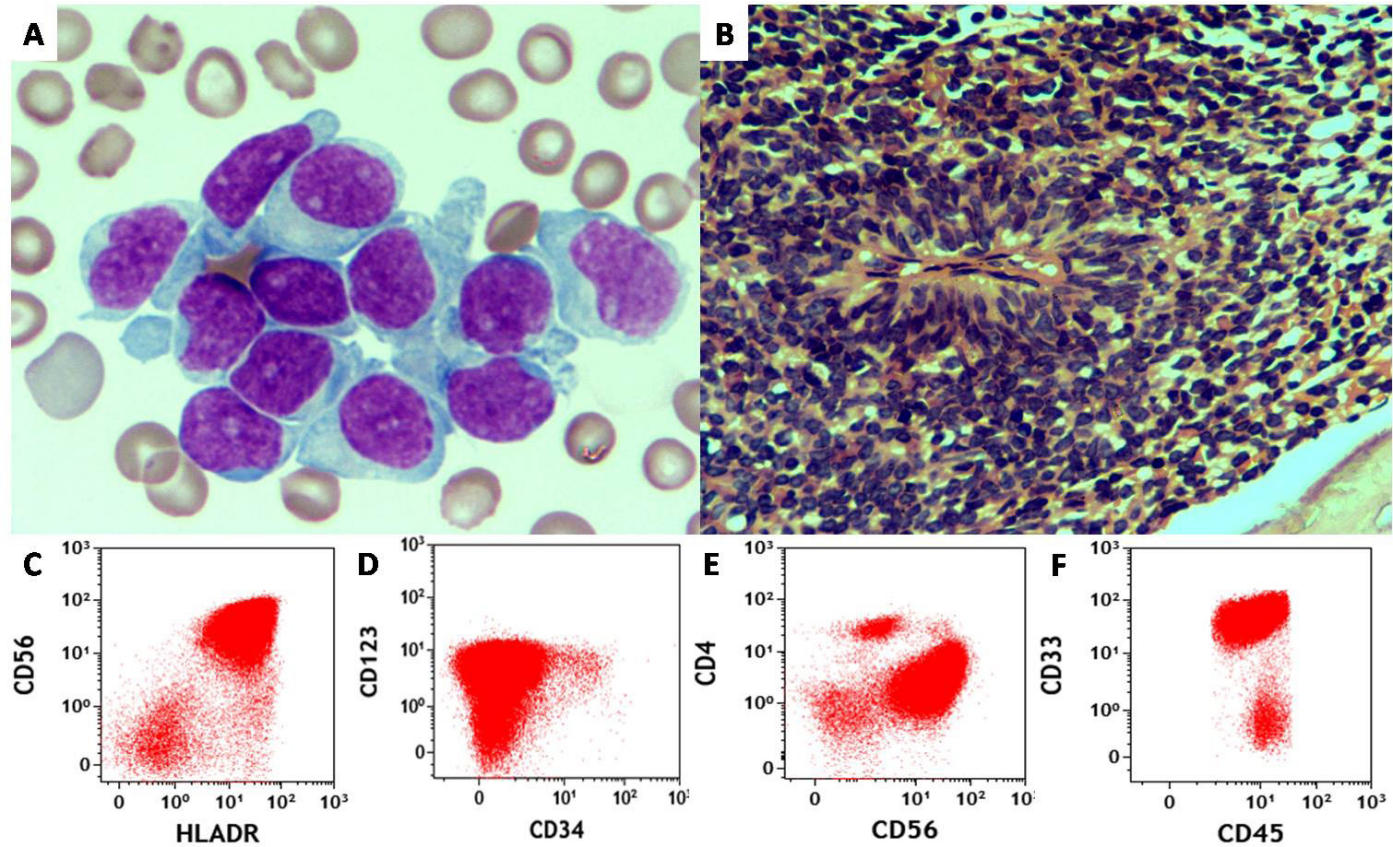
A) Bone marrow aspirate smear showing blastoid cells (Giemsa stain, 1000^x^). B) Bone marrow biopsy showing pseudo-rosette formation by tumor cells (hematoxylin and eosin stain, 400^x^). C-F) Flow cytometric dot plots showing characteristic surface immunophenotypic marker expression profile of blastic plasmacytoid plasma cell neoplasm.

We read the recent article on blastic plasmacytoid dendritic cell neoplasm (BPDCN) [1] with interest and would like to add another interesting case to the list with an intriguing finding in the bone marrow. A 64-year-old male patient presented with complaints of fever and easy fatigability. Moderate pallor and hepatosplenomegaly were present. Laboratory investigations showed pancytopenia with hemoglobin of 72 g/L, total leukocyte count of 0.9x10^9^/L, and platelet count of 45x10^9^/L. Bone marrow examination revealed 92% medium-sized blastoid cells with fine chromatin, inconspicuous nucleoli, and agranular cytoplasm with pseudopodia (Figure 1A). Myeloperoxidase and periodic acid-Schiff staining was negative. Bone marrow biopsy showed sheets of tumor cells forming peculiar pseudo-rosette formations at places (Figure 1B). Immunophenotyping identified a dim CD45-positive blast population, which was also positive for CD4, CD56, CD123, CD33, CD38, and HLA-DR. The blasts were negative for CD34, B-cell markers, T-cell markers (surface and cytoplasmic CD3, CD4, CD8, CD5, and CD7), myeloid markers (CD13, CD117, and myeloperoxidase), monocytic markers (CD14, CD64, and CD36), and markers of immaturity (TdT, CD34) (Figures 1C-1F). The patient was diagnosed with blastic plasmacytoid dendritic cell neoplasm (BPDCN). His general condition deteriorated and he died due to progressive illness.

BPDCN is a rare and aggressive hematological malignancy associated with a high frequency of skin and/or bone marrow infiltration and leukemic dissemination [2]. Absence of lineage-specific markers in association with high levels of expression of plasmacytoid dendritic cell markers such as CD123, CD4, CD56, and CD45RA is pathognomonic of BPDCN [2]. Rosette formation by hematopoietic neoplasms is known [3,4]; however, this is a unique case of pseudo-rosette formation by BPDCN in bone marrow, emphasizing that hematopathologists should consider BPDCN in the differential diagnosis of pseudo-rosette-forming tumors in the bone marrow.
