# Supplementary material for: Pseudo-Rosette-Forming Blastic Plasmacytoid Dendritic Cell Neoplasm
Source: Turk J Haematol. 2020 Aug 28;37(3):210–1. doi: 10.4274/tjh.galenos.2020.2020.0141 (PMC7463204; doi:10.4274/tjh.galenos.2020.2020.0141)
Supplement: Related texts

# Reply

## To the Editor,

We have read the letter “Pseudo-Rosette-Forming Blastic Plasmacytoid Dendritic Cell Neoplasm,” which presents a very fine example of blastic plasmacytoid dendritic cell neoplasia (BPDCN).

There is one published case in the literature of CD4+ and CD56+ blastic NK cell lymphoma with pseudo-rosettes [1]. Although this case was diagnosed with a name other than BPDCN, the clinical, morphological, and immunophenotypic features are very similar to BPDCN, except for TdT positivity. BPDCN is a relatively new name and blastic NK cell lymphoma was one of BPDCN’s older names before BPDCN was defined and accepted as a new and separate entity [2].

Pseudo-rosette formation is a very well-known infiltration pattern for many lymphomas, especially central nervous system lymphomas, T/NK cell lymphomas, etc. However, except for this particular case, even in larger case series, pseudo-rosettes are not defined as a common or a diagnostic pattern for BPDCN [3,4]. We did not see pseudo-rosettes in our small case series of BPDCN [5]. The patterns of the tumors in our series were mostly diffuse in the dermis in skin biopsies and interstitial in bone marrow biopsies, as expected.

Since we do not have enough information about this very rare tumor, diagnostic and treatment options are very limited and controversial. Therefore, it is very valuable to learn any features of this rare entity.

**Best Regards,**

Ahu Senem Demiröz, Cuyan Demirkesen, Ayşe Salihoğlu, Nükhet Tüzüner

## References

[1] Ko YH, Kim SH, Ree HJ (1998). Blastic NK-cell lymphoma expressing terminal deoxynucleotidyl transferase with Homer-Wright type pseudorosettes formation. Histopathology.

[2] Swerdlow SH, Campo E, Harris NL, Jaffe ES, Pileri SA, Stein H, Thiele J (2017.). (eds). WHO Classification of Tumours of Haematopoietic and Lymphoid Tissues. 4th ed. Lyon, International Agency for Research on Cancer.

[3] Julia F, Dalle S, Duru G, Balme B, Vergier B, Ortonne N, Vignon-Pennamen MD, Costes-Martineau V, Lamant L, Dalac S, Delattre C, Déchelotte P, Courville P, Carlotti A, De Muret A, Fraitag S, Levy A, Mitchell A, Petrella T (2014). Blastic plasmacytoid dendritic cell neoplasms: clinico-immunohistochemical correlations in a series of 91 patients. Am J Surg Pathol.

[4] Pagano L, Valentini CG, Grammatico S, Pulsoni A (2016). Blastic plasmacytoid dendritic cell neoplasm: diagnostic criteria and therapeutical approaches. Br J Haematol.

[5] Demiröz AS, Demirkesen C, Salihoğlu A, Tüzüner N (2020). Blastic plasmacytoid dendritic cell neoplasia: a single center experience. Turk J Hematol.
